# Deep Prediction Model Based on Dual Decomposition with Entropy and Frequency Statistics for Nonstationary Time Series

**DOI:** 10.3390/e24030360

**Published:** 2022-03-02

**Authors:** Zhigang Shi, Yuting Bai, Xuebo Jin, Xiaoyi Wang, Tingli Su, Jianlei Kong

**Affiliations:** 1 School of Artificial Intelligence, Beijing Technology and Business University, Beijing 100048, China; shizhigang@st.btbu.edu.cn (Z.S.); wangxy@btbu.edu.cn (X.W.); sutingli@btbu.edu.cn (T.S.); kongjianlei@btbu.edu.cn (J.K.); 2 Beijing Laboratory for Intelligent Environmental Protection, Beijing Technology and Business University, Beijing 100048, China; 3 State Environmental Protection Key Laboratory of Food Chain Pollution Control, Beijing Technology and Business University, Beijing 100048, China

**Keywords:** time series prediction, deep learning, variational mode decomposition, feature extraction

## Abstract

The prediction of time series is of great significance for rational planning and risk prevention. However, time series data in various natural and artificial systems are nonstationary and complex, which makes them difficult to predict. An improved deep prediction method is proposed herein based on the dual variational mode decomposition of a nonstationary time series. First, criteria were determined based on information entropy and frequency statistics to determine the quantity of components in the variational mode decomposition, including the number of subsequences and the conditions for dual decomposition. Second, a deep prediction model was built for the subsequences obtained after the dual decomposition. Third, a general framework was proposed to integrate the data decomposition and deep prediction models. The method was verified on practical time series data with some contrast methods. The results show that it performed better than single deep network and traditional decomposition methods. The proposed method can effectively extract the characteristics of a nonstationary time series and obtain reliable prediction results.

## 1. Introduction

A time series is a significant representation of various objects and systems that describes their changing processes. The prediction of time series aims at estimating their future trends with hidden characteristics in the historical data. Time series prediction has gained widespread attention in many fields, such as meteorology [1,2], the stock market [3], environment pollution control [4,5], and data mining on the Internet. The reliable prediction of future trends can help administrators in comprehensive and scientific decision making [6]. At the core of making predictions is an appropriate model based on the features of the data. Predicting a time series effectively has been a hot issue in the fields of data mining and machine learning.

The early methods of time series prediction are based on statistical theory [7,8,9], which uses statistical analysis to model time series. Statistical methods perform well on stationary series, but they usually fail when the data comprises complex noises and nonstationary trends. Shallow neural networks [10] have been widely used due to their self-learning ability. The research shows that shallow networks face overfitting and insufficient fitting problems in different data conditions. Deep networks [11] were developed in view of the network structure and data scale. They can fit data trends based on the inner multiple layers that help model high-dimensional nonlinear relationships. Deep networks also benefit from mass data, which can train models with vast iterations. Although they have powerful learning abilities, deep networks may fail on nonstationary data with unpredictable noise and complex features.

For nonstationary time series data with complex noise, studies have been conducted to decompose the data for the feature extraction, such as seasonal-trend decomposition procedure based on LOESS (STL), wavelet decomposition (WD), empirical mode decomposition (EMD), and ensemble empirical mode decomposition (EEMD). Variational mode decomposition (VMD) [12,13,14] improves the mode aliasing and noise sensitivity of the existing method. It can be regarded as a relatively robust decomposition method, and it has been proved valid in practical data analysis. In VMD, the number of decomposition layers can be preset to effectively separate different frequency components, which is more widely applicable to nonstationary data. However, some practical issues occur in the VMD method, including the determination of the components and the inadequate decomposition of the subsequence. The degree of the decomposed subsequences directly influences the feature extraction. For data with implicit trends and noise, the complexity of the decomposition components is still very high. The ideal simple components cannot be obtained by a single decomposition. The question of how to obtain the subsequence with clear trend features and without complex noise has been a major issue.

Considering the feature extraction ability of VMD and the prediction performance of deep networks, an integrated model is proposed in this paper. Nonstationary time series data are the main object of analysis, and a deep prediction model with dual decomposition is proposed. In the dual decomposition, a new method was designed to determine the number of VMD subsequences based on information entropy. The requirements of the dual decomposition were determined with the frequency characteristics of the components. Finally, a general framework was built, which consists of a dual VMD and a gated recurrent unit (GRU). Experiments were conducted to show that the proposed method can extract features from time series data with an automatic mechanism and achieve reliable prediction results.

This paper is organized as follows. Section 2 introduces the related works on time series prediction. Section 3 presents the main prediction method, including the decomposition mechanism and the deep neural networks. Section 4 presents the experiments. The experimental results are discussed in Section 5. The work is concluded in Section 6.

## 2. Related Works

### 2.1. Prediction Methods Based on Machine Learning

As mentioned in Section 1, research on time series prediction has gained widespread attention. The methods can be classified into three categories, namely statistical models, shallow neural networks, and deep networks. In this section, the related works are introduced, with a focus on machine learning methods, especially deep networks.

Statistical models describe changes in time series data using mathematical analysis. The ARMA [15] model is a typical statistical model that combines the AR and MA models. The ARIMA [16] model adds differential processing based on the ARMA model and can be used to process nonstationary data. In addition, the GARCH [17] and state space methods, represented by Kalman filters, [18,19] are used to build predictive models.

Artificial neural networks, such as the BP, Elman, and RBF, can autonomously capture nonlinear features in data. Various methods have been studied to solve practical problems. Xue et al. [20] used extreme learning machines to study financial time series, and Lin et al. [21] established the support vector machine method to predict electricity generation. Amjady et al. [22] constructed a fuzzy neural network method to predict electricity prices. Yang et al. [23] proposed the dynamic regularized echo state network (DRESN) model, which can dynamically determine the structure of an echo state network (ESN) network. Compared to ESN networks, DRESN networks have better accuracy.

In recent years, neural networks with deeper layers have been used to build models. Che et al. [24] built the RNN network to predict multivariate time series. Based on the RNN network, Hochreiter et al. [25] proposed a long short-term memory (LSTM) network to effectively solve the problems of vanishing gradients and the long-term dependence of RNN networks. Fischer et al. [26] used the LSTM network for financial market prediction in their research, and LSTM is superior to memoryless classification methods, such as random forest, deep neural networks, and logistic regression classifier. As an improvement to LSTM, GRU [27] reduces the gating cycle unit based on LSTM, thereby reducing the number of parameters. While obtaining the same or even better results, the calculation speed is increased. Ding et al. [28] used the GRU network to predict short-term wind speed.

Statistical models are appropriate for time series with obvious trends. Machine learning methods supplement statistical models for their lack of nonlinear fitting ability. In the existing literature and previous experimental studies, it has been shown that deep networks still need to improve the fitting of nonstationary time series with noise. It is possible to improve the prediction accuracy by introducing data pre-analysis before deep network training.

### 2.2. Decomposition and Prediction Methods

For the feature extraction of complex time series data, decomposition methods have been applied widely. In addition, decomposition has been combined with machine learning to solve the problem of time series prediction. The aim of these methods is to decompose raw data into multiple subsequences, and appropriate machine learning models have been established according to the characteristics of different subsequences.

Various decomposition methods have been studied to extract data features. The seasonal-trend decomposition procedure based on LOESS (STL) [29,30] decomposes time series into three components—period, trend, and residual. Wavelet decomposition (WD) [31] improves the Fourier transform method. An original series is decomposed into a series of wavelets by translating and scaling the wavelets. Empirical mode decomposition (EMD) [32] obtains an inherent mode function without a pre-set basis function. Ensemble empirical mode decomposition (EEMD) [33,34] can distribute a series into appropriate reference scales by introducing white noise. Although they have the ability of feature extraction, there are shortcomings in these methods. STL is generally applied to periodic time series with stable trends. It is difficult for WD to choose a suitable wavelet base. EMD has problems with mode aliasing and noise sensitivity, and EEMD generates a lot of redundant information during the decomposition process.

The integrated prediction methods have been applied based on decomposition. Jin et al. [35] decomposed data into period, trend, and residual components by STL. For the period and residual components, GRU networks were built to predict the values. The trend component has good linear characteristics, so the ARIMA model, which has a good predictive ability for linear systems, was selected to build the prediction model. The prediction results of the three models were integrated into the final result. Compared to the results of a single GRU model and all three components using the GRU network, the proposed model achieved the best performance. In addition, Jin et al. [36] used EMD to divide the original data into intrinsic mode functions (IMFs) with different frequency characteristics. They used the convolutional neural network to extract local feature components to build a classification model and reconstructed components with similar frequency characteristics into a group. All components were divided into three groups, and each group was modeled by the GRU network. Their method solved the shortcomings of the uncertain layers of EMD and achieved the best accuracy compared to other multiple-component combinations. Niu et al. [37] proposed a hybrid model based on evolutionary extreme learning machine and VMD to predict annual runoff time series. First, the VMD method was used to decompose the original stream into a series of components. Secondly, each component was predicted by constructing an appropriate extremum learning machine model, and the model parameters were adjusted using the gravity search algorithm. Finally, the prediction results of all the models were aggregated and used as the simulation output. Yang et al. [38] established a hybrid method combining wavelet transform, kernel extreme value learning machine (KELM) based on adaptive particle swarm optimization, and ARMA. An adaptive particle swarm optimization algorithm (SAPSO) was used to find the best kernel parameters. After testing the wavelet decomposition components, the ARMA model was applied to predict stationary sequences as a new input set, and the SAPSO-KELM model was constructed to predict nonstationary sequences. The experimental results showed that the method has more accurate prediction ability, better versatility, and practicability than the single method and other hybrid methods. Xie et al. [13] constructed a comprehensive model to predict solar output power combining VMD, ARMA, and a deep belief network (DBN). The time series were decomposed into components of different frequencies by VMD, and then the DBN and ARMA were established to predict high-frequency components and low-frequency components, respectively. Finally, the predicted values were reconstructed to get the final result. The hybrid method was superior to the single prediction model, proving its good accuracy and reliability.

**Figure 1:**
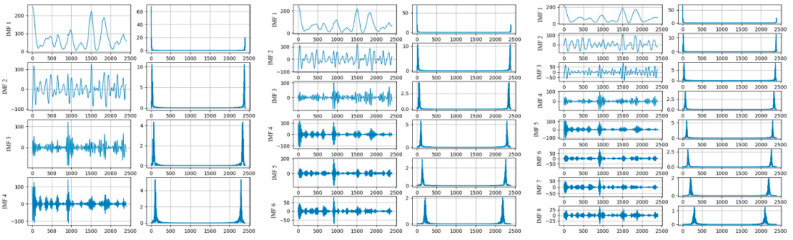
The VMD decomposition results and spectrum of different layers.

As mentioned above, the decomposition methods are not consummate, and VMD is regarded as a relatively effective solution. In previous studies, we applied the VMD method to PM 2.5 monitoring data analysis. PM 2.5 concentration data in Beijing were recorded every hour [39]. The data from 100 days were selected for analysis, starting on 1 January 2016, with a total of 2400 h. The PM 2.5 concentration data were decomposed with VMD, in which the different decomposition layers were tested. The decomposition results are shown in Figure 1.

The subsequence and the frequency distribution after decomposition are shown in Figure 1, where the data are decomposed into 4, 6, and 8 layers, respectively. The center frequencies of the subsequences after the four-layer decomposition were 3.49, 23.14, 65.57, and 107.51. After six layers of decomposition, the center frequencies of the subsequences were 3.33, 21.79, 58.24, 100.42, 139.43, and 208.38. The center frequencies of the subsequences obtained after the eight-layer decomposition were 2.63, 16.46, 34.22, 68.45, 102.15,139.67, 203.53, and 294.96. It can be seen that the frequencies of the subsequences increased along with the decomposition layers. Subsequences with higher frequencies had better periodicity, which is easier to model and can improve the prediction accuracy.

Two practical problems can be seen in the analysis above. First, the number of subsequences impacted the feature complexity, but it was usually determined by artificial experience. An automatic mechanism should be explored for the determination of the subsequence quantity. Second, some subsequences still had higher center frequencies, which negatively impacted the prediction modeling. The complex subsequences should be decomposed more than once, and criteria should be determined for which subsequences should be decomposed again.

In this study, we mainly investigated deep networks with data feature extraction for nonstationary time series, which is significantly different from previous studies. Our innovative contributions are highlighted as follows:

1. An automatic mechanism was designed for the decomposition process of VMD, in which the criteria are determined based on the entropy and frequency to determine the number of subsequences and the dual decomposition parts.
2. A general framework was constructed to integrate the dual decomposition mechanism and deep networks for time series prediction. The integrated deep model effectively solves the prediction issue with nonstationary time series.

## 3. Deep Prediction Model with Dual Decomposition

### 3.1. Dual Decomposition Criteria in VMD

#### 3.1.1. Decomposition Method of VMD

VMD is an adaptive signal processing method with a completely non-recursive mechanism. It decomposes the input signal into several IMFs with limited bandwidth. In the optimization process, each component can be compressed around a central frequency to achieve the separation of inherent modal components. It overcomes the problems of end effects and modal component aliasing in the EMD method. For time series data with high complexity and strong nonlinearity, the VMD method can reduce the non-stationarity to obtain relatively stationary subsequences. For the original signal, the corresponding constrained variational model is expressed as

(1) $$\frac{\min}{\left\{u_{k}\},\{w_{k}\right\}}=\left\{\sum_{k}||\partial_{t}\left[\left(\delta (t)+j/\pi t)*u_{k}(t\right)\right]e^{-jw_{k}t}{\left|\right|}_{2}^{2}\right\}$$

(2) $$s.t.\sum_{k=1}^{k}u_{k}=f$$

where k is the number of modals to be decomposed. $u_{k}$, $w_{k}$ are the k-th modal component and center frequency after decomposition, respectively. δ(t) is the Dirac function, and ∗ is the convolution operator. The Lagrange multiplication operator λ is introduced to transform the constrained variational problem into an unconstrained variational problem. The augmented Lagrange expression is as follows:

(3) $${\hat{u}}_{k}^{n+1}(w)\leftarrow \frac{\hat{f}(w)-\sum_{i\neq k}{\hat{u}}_{i}(w)+\hat{\lambda }(w)/2}{1+2\alpha {\left(w-w_{k}\right)}^{2}}$$

(4) $$\omega_{k}^{n+1}\leftarrow \frac{\int_{0}^{\infty }\omega {\left|{\hat{u}}_{k}^{n+1}(\omega )\right|}^{2}d\omega }{\int_{0}^{\infty }{\left|{\hat{u}}_{k}^{n+1}(\omega )\right|}^{2}d\omega }$$

(5) $${\hat{\lambda }}^{n+1}(\omega )\leftarrow {\hat{\lambda }}^{n}(\omega )+\gamma \left(\hat{f}(\omega )-\sum_{k}{\hat{u}}_{k}^{n+1}(\omega )\right)$$

where γ is the noise tolerance, which satisfies the fidelity requirements of signal decomposition, and ${\hat{u}}_{k}^{n+1}(w)$, ${\hat{u}}_{i}(w)$, $\hat{f}(\omega )$, and $\hat{\lambda }(w)$ are the Fourier changes of $u_{k}^{n+1}(t)$, $u_{i}(t)$, f(t), and λ(t), respectively. 

The final decomposition results are obtained by the following steps. First, the parameters ${\hat{u}}_{k}^{1}$, $\omega_{k}^{1}$, $\lambda^{1}$ and N are initialized. Then, the parameters are updated until $\sum_{k}{\left\|{\hat{u}}_{k}^{n+1}-{\hat{u}}_{k}^{n}\right\|}_{2}^{2}/{\left\|{\hat{u}}_{k}^{n}\right\|}_{2}^{2}<\varepsilon$ is met. Finally, the original signal f can be decomposed to *K* IMFs. The algorithm of VMD is shown as the following Algorithm 1:
**Algorithm 1: VMD**For k=1:K
 Initialization ${\hat{u}}_{k}^{1},\;\omega_{k}^{1},\;\lambda^{1}\;\mathrm{and}\;\mathrm{the}\;\mathrm{maximum}\;\mathrm{number}\;\mathrm{of}\;\mathrm{iterations}\;N.$
   For *n* = 1; *n* < *N*; *n*++;      Update ${\hat{u}}_{k}^{n+1}(w),\;\omega_{k}^{n+1},\;{\hat{\lambda }}^{n+1}(\omega ).$      If $\sum_{k}\|{\hat{u}}_{k}^{n+1}-{\hat{u}}_{k}^{n}{\|}_{2}^{2}/{\left\|{\hat{u}}_{k}^{n}\right\|}_{2}^{2}<\varepsilon :$       break;
End ForEnd For

#### 3.1.2. Criteria for the Number of Components

The decomposition *K* number is a significant parameter in the Algorithm 1. Modal aliasing may occur when the *K* is large, and the original data cannot be decomposed effectively when the *K* is small. In practice, the number of components after the decomposition is determined artificially based on personal experience. The subjective judgment may not apply to the current condition. Moreover, the number determined cannot be generalized when the data trends and features change. An automatic mechanism should be explored to determine the number of decomposed components. The criteria are proposed based on the analysis of feature changes with information entropy.

The original time series data are decomposed to form the component set of IMFs. In the decomposition process, the IMF is marked as $u_{k}$, the center frequency of which is $w_{k}$. The center frequency reflects the nonstationary characteristics of the decomposed IMF. The IMFs are approximate when their frequencies are similar to low gradients, which may lead to modal aliasing. The change in the center frequency can be considered the terminal condition of the decomposition. Information entropy is introduced to measure the change rate of the center frequency. The information entropy $e_{k}$ of the existing IMFs is calculated as follows:

(6) $$e_{k}=-\sum_{k=1}^{n}p(w_{k})\log(p(w_{k}))$$

where p is the probability measure of the object data. Based on the criteria, the probability can be concerted from the normalization, as follows:

(7) $$p(w_{k})=\frac{w_{k}}{\sum_{i=1}^{w-1}w_{i}}$$

The change rate of $e_{k}$ is obtained as

(8) $$d_{k}=\left|e_{k}-e_{k-1}\right|$$

**Figure 2:**
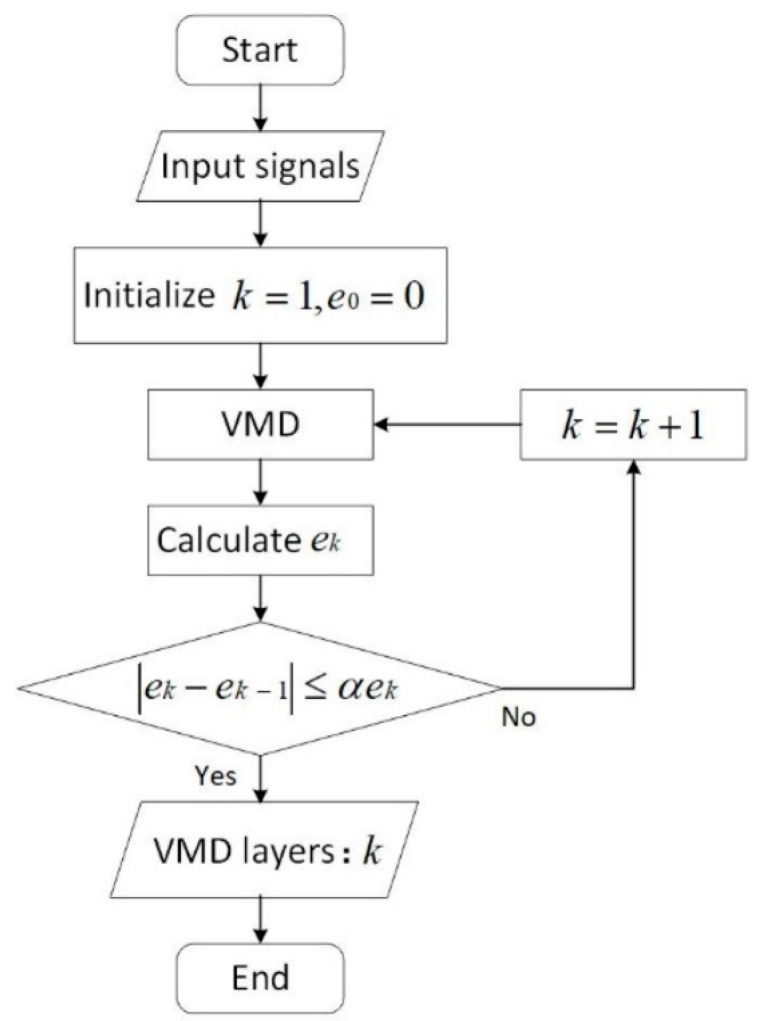
Flowchart of the determination of the decomposed component amount.

The change rate of the information entropy $d_{k}$ is considered to be the criteria. The decomposition of VMD ends if $d_{k}\leq \alpha e_{k}$, where α is an adjusting parameter that is usually set between 0.05 and 0.2 following the Pareto rule. The determination of the component amount can be conducted as shown in the flowchart in Figure 2.

#### 3.1.3. Criteria for Dual Decomposition

For prediction based on decomposition, the complexity degree of the decomposed component impacts the prediction difficulty level and precision. For IMFs obtained from original data, some still have high nonlinear and nonstationary trends. The complex IMFs with low center frequencies need further decomposition, which is the dual decomposition method of this study. 

**Figure 3:**
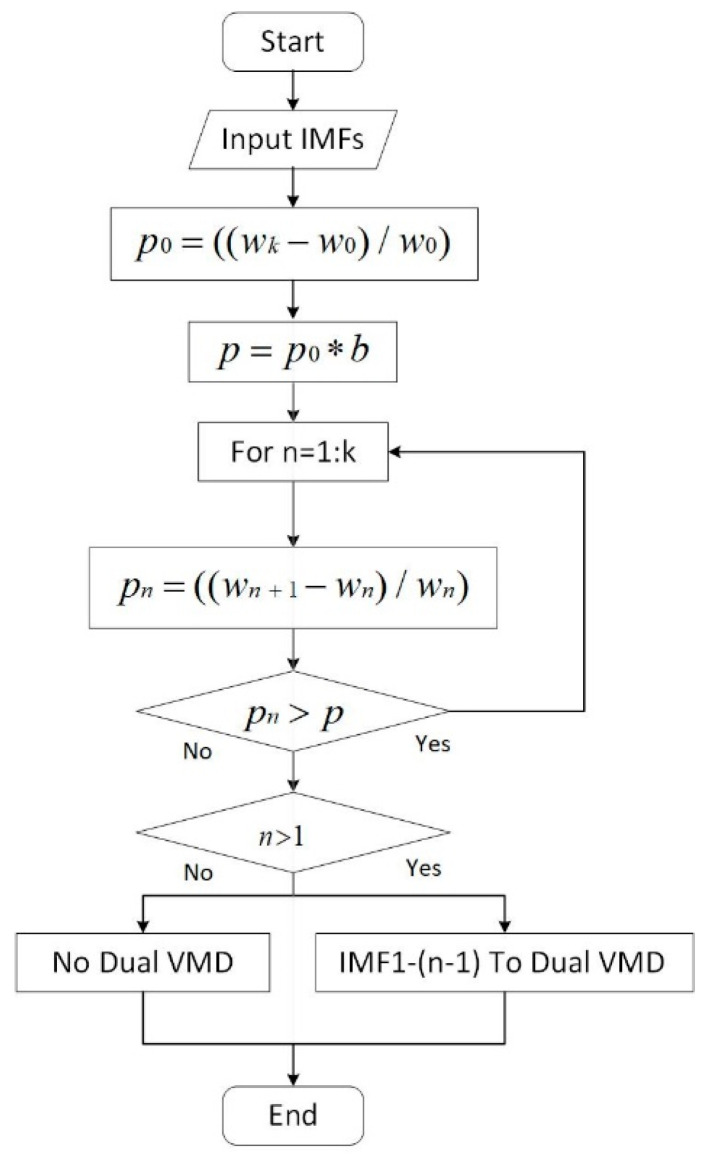
Flowchart of dual decomposition for low-frequency components.

When the center frequency of an adjacent IMF changes greatly, the decomposition is insufficient and it needs to be decomposed again. The flowchart of the dual decomposition is shown in Figure 3.

As shown in Figure 3, first, the change rates of the center frequencies of the first and last IMF are defined as *p*_0_ and $p=p_{0}\times b$, respectively. (0<b<1) is defined as the second decomposition threshold. Then, starting from the first subcomponent, the rate of change between the *n*-th IMF and the (*n* + 1)-th IMF center frequency is calculated as $p_{n}$. When $p_{n}$ is greater than the threshold, it indicates that the data in this frequency range is not sufficiently decomposed and a second decomposition is required. However, when $p_{n}$ is less than the threshold value for the first time, it indicates that the latter high-frequency components have been decomposed completely in the first VMD and there is no need to continue with a second decomposition. All the low-frequency components that need to be decomposed are divided into three components again. When the rate of change of all adjacent components obtained from the first decomposition is less than the threshold, dual decomposition is not required. 

### 3.2. Deep Prediction Model

#### 3.2.1. Basic Deep Network of GRU

**Figure 4:**
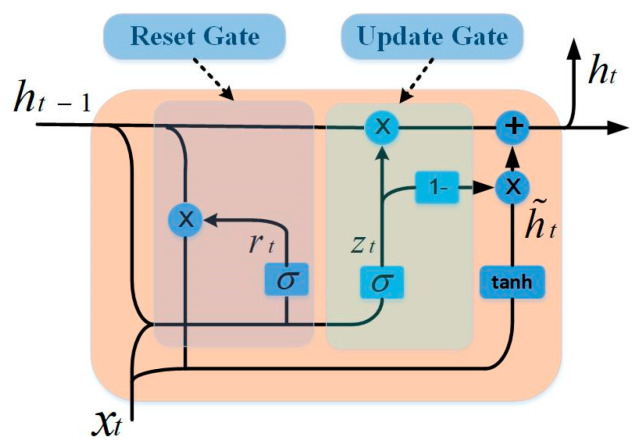
The GRU cell structure.

GRU is a recurrent neural network that updates and optimizes weights by gradient descent algorithms. With its delicate network characteristics and structure, it achieves good performance in the prediction of nonstationary time series. The GRU contains two gating units—the reset gate and the update gate. The reset gate is used to ignore information in the current state. The smaller the value, the more the information is ignored. The update gate is used to record the information of the current state. The larger the value, the more the information is recorded in the current state. In our method, the GRU network is used to construct a prediction model for each subsequence. Figure 4 shows the internal structure of the GRU.

For a GRU cell, the relationship between its internal parameters can be expressed by Equation (6), as follows:

(9) $$\begin{array}{l} r_{t}=\sigma (W_{rx}x_{t}+W_{rh}h_{t-1}+b_{r})\\ u_{t}=\sigma (W_{ux}x_{t}+W_{uh}h_{t-1}+b_{u})\\ {\tilde{h}}_{t}=\tanh(W_{\tilde{h}x}x_{t}+W_{\tilde{h}r}(r_{t}\circ h_{t-1})+b_{\tilde{h}})\\ h_{t}=(1-u_{t})\circ h_{t-1}+u_{t}\circ {\tilde{h}}_{t}\\ y_{t}=\sigma (W_{y}h_{t}+b_{y}) \end{array}$$

where $r_{t}$, $u_{t}$, ${\tilde{h}}_{t}$, $h_{t}$, and $y_{t}$ represent reset gate, update gate, hidden state, output state, and output respectively. $W_{rx}$, $W_{rh}$, $W_{ux}$, $W_{uh}$, $W_{\tilde{h}x}$, $W_{\tilde{h}r}$, and $W_{y}$ represent the weight matrix of training. $b_{r}$, $b_{u}$, $b_{\tilde{h}}$, and $b_{y}$ represent the bias. σ and tanh represent the activation function. ∘ represents multiplying elements.

#### 3.2.2. Framework of the Deep Prediction Model with Dual Decomposition

**Figure 5:**
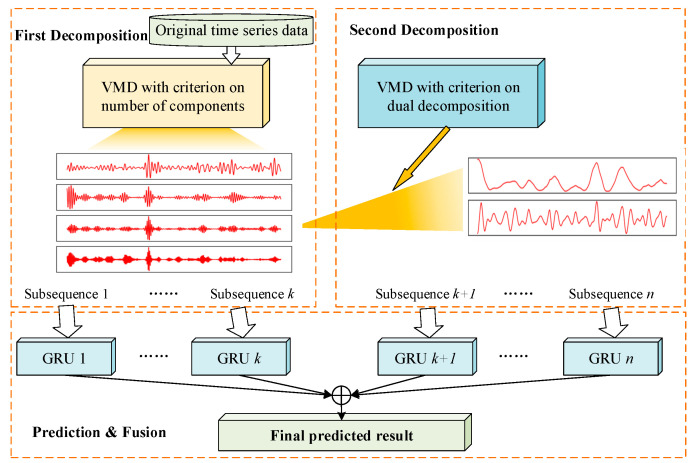
Framework of the deep prediction method with dual decomposition.

The framework for a deep prediction model with dual decomposition is proposed in this paper. It consists of the modules of a first decomposition, second decomposition, and prediction and fusion. The general framework design is shown in Figure 5.

As shown in Figure 5, the proposed model includes three parts—first decomposition, second decomposition, and prediction and fusion. In the module of the first decomposition, the original data are decomposed into IMFs with the VMD method, in which the criteria are set to determine the number of components. The first decomposition ends when the frequency variation meets the conditions following the calculation in Section 3.1.2. For IMFs with high nonlinear and nonstationary features, they are processed with a second decomposition. The criteria in Section 3.1.3 guide the determination and selection of the components to be decomposed again. Finally, in the prediction and fusion module, the decomposed subsequences are predicted with GRUs. The prediction results of all individual network models are fused to obtain the final output.

## 4. Experiment and Result

### 4.1. Experimental Setting and Data Set

Experiments were designed and carried out to verify the proposed method. For the prediction model, a practical data set was used to test its performance. The data of PM 2.5 concentration mentioned in Section 2.2 were selected as the practical data.

For the data totaling 2400 h, the first 80% of the data were used as the training set to build the model, and the other 20% of the data were used to test the accuracy of the model. The experimental platform was built on 64-bit Windows. The RAM was 8 GB, and the core was i7-8565u with 1.8 GHz. The program used Python version 3.7.4. The deep learning framework used the Tensorflow interface of Keras, and the optimizer was Adam.

The aim proposed method is to solve the problem of data decomposition, including determining the number of components and which ones should be decomposed again. Three sets of tests were conducted to verify the different aspects of the proposed method.

Test 1: The criteria for the number of decomposed components were tested. The original data were analyzed with the method described in Section 3.1.2 to determine the optimal number of IMFs. Then, the data were decomposed with different layers following the exhaustion method. The calculated and exhaustive results were analyzed.

Test 2: The effect of the dual decomposition on the prediction was tested. For the decomposed components in Test 1, parts of them still had complex features. The criteria were set to determine which components should be decomposed again. The prediction performance of the dual decomposition was analyzed.

Test 3: The proposed method was tested and compared to other methods. As discussed in Section 2, five well-known models were used to verify our method, including RNN, LSTM, GRU, decomposition-ARIMA-GRU-GRU [35], and EMDCNN-GRU [36], which are introduced briefly as follows.

RNN, LSTM, and GRU are typical machine learning methods that have been widely used in time series prediction. RNN establishes weighted connections between neurons in layers. LSTM and GRU were developed from RNN by introducing different kinds of gates to enhance information storage and screening.

Decomposition-ARIMA-GRU-GRU and EMDCNN-GRU are proposed as advanced approaches based on machine learning models. They mainly focus on the decomposition of the original data to reduce complexity. The decomposition is executed in a single pass, which is different from the dual decomposition presented in this paper.

To evaluate the performance of the model, the following metrics were used:

(10) $$RMSE=\sqrt{\frac{1}{T}{\sum_{i=1}^{T}\left(y_{r}-y_{p}\right)}^{2}}$$

(11) $$MAE=\frac{\sum_{i=1}^{T}\left|y_{r}-y_{p}\right|}{T}$$

(12) $$R^{2}=1-\frac{\sum_{i=1}^{T}{\left(y_{p}-y_{r}\right)}^{2}}{\sum_{i=1}^{T}{\left(y_{p}-y_{rv}\right)}^{2}}$$

(13) $$CC=\frac{\sum_{i=1}^{T}\left[y_{r}(i)-y_{rv}(i)\right]\left[y_{p}(i)-y_{pv}(i)\right]}{\sqrt{{\sum_{i=1}^{T}\left[y_{r}(i)-y_{rv}(i)\right]}^{2}}\sqrt{{\sum_{i=1}^{T}\left[y_{p}(i)-y_{pv}(i)\right]}^{2}}}$$

(14) $$AE=y_{p}-y_{r}$$

where $y_{r}$ represents the real value, $y_{p}$ represents the predicted value, T represents the number of data, $y_{rv}$ represents the average value of the real value, and $y_{pv}$ represents the average value of the predicted value. The root mean squared error (RMSE) measures the deviation between the predicted value and the true value. The smaller the value of the RMSE, the better the model prediction. The mean absolute error (MAE) is used to measure the error between the observed value and the real value. When the error is zero, it is a perfect model. The square of R is between 0 and 1. The closer it is to 1, the more accurate the model is. CC is the correlation coefficient [39] and represents a linear relationship between the predicted and real values. The closer it is to 1, the stronger the linear relationship is.

### 4.2. Results

#### 4.2.1. Test on the Number of Decomposition Components

Different decomposition layers significantly influence the prediction results. The algorithm for the criteria proposed in Section 3.1.2 was applied to obtain the number of decomposed layers, and the calculation result was 6. In the first test, the original data were decomposed following the exhaustion method, in which data are decomposed into 3–12 layers. The decomposed IMFs were predicted with GRUs and the fusion result was finally obtained. The RMSEs of the prediction results of different decomposition layers are listed in Table 1. The calculation time spent for each experiment is also presented.

**Figure 6:**
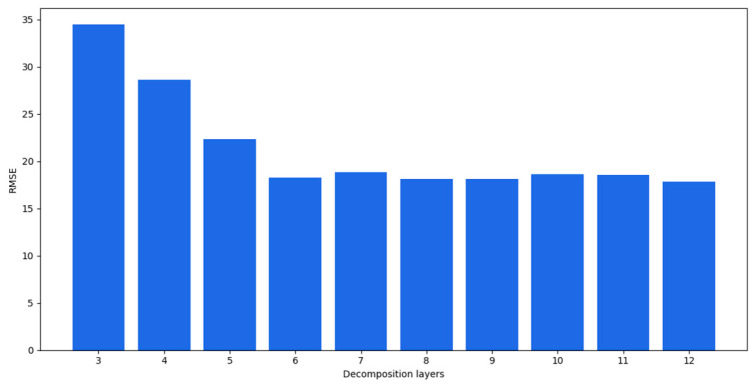
RMSEs of prediction results for different decomposition layers.

As the number of decomposition layers increased, the accuracy of the model increased. When the number of decomposition layers reached six, the model performance tended to be stable. Although the 8-, 9-, and 12-layer decomposition models performed better than the six-layer decomposition, the time spent increased significantly. The changes in the RMSEs are shown in Figure 6. The horizontal axis is the number of decomposition layers and the vertical axis is the RMSE.

As shown in Figure 6, the prediction performance got better as the number of decomposition layers increased and was between three and six layers. When the number of decomposition layers was greater than 6, the prediction accuracy tended to be stable. As mentioned at the beginning of Section 4.2.1, it was observed that the exhausted results met the calculated values, where the optimal number of decomposed IMF layers was six.

#### 4.2.2. Test of Dual Decomposition

**Figure 7:**
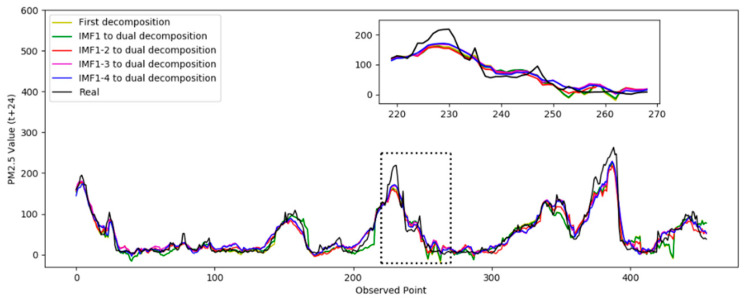
Comparison of the prediction results for PM 2.5.

**Figure 8:**
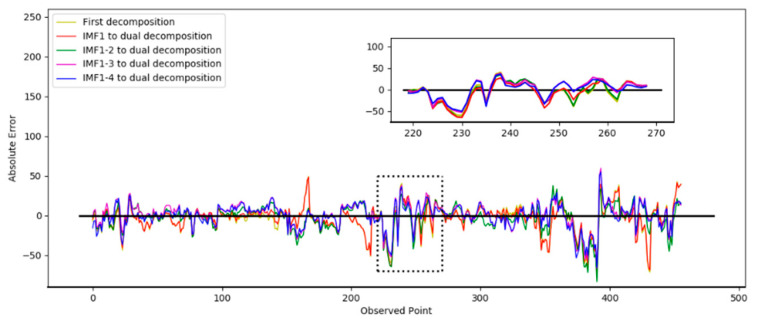
Absolute errors of prediction results in different decomposition situations.

In Test 1, the optimal number of decompositions was determined to be six. In the previous analysis, the low-frequency subsequence still had highly complex trends. Based on the criteria in Section 3.1.3, the third to the sixth IMFs still had obvious nonstationary features. Then, the dual composition was applied to the IMFs. In the test, the different numbers of IMFs were decomposed again to verify the performance. In the following results, “IMF 1” means only the first IMF was decomposed twice, “IMF 1–2” means the first and second IMFs were decomposed twice, “IMF 1–3” means the first to the third, and “IMF 1–4” means the first to the fourth. The prediction results based on the different decomposition situations are shown in Figure 7. The absolute errors of the prediction results are shown in Figure 8.

Figure 7 shows the prediction results from the second decomposition. The black curve represents the real result. It was observed that the prediction curve of IMF 1–4 was closer to the true value, which indicates the best performance. Figure 8 shows the absolute error curve calculated by Equation (11). The black straight line represents the standard line corresponding to the true value. The closer it is to the black line indicates the prediction error of the model was relatively minimal and the performance was better. It can be seen that the dual decomposition of IMF 1–4, represented by the blue curve, is closest to the black curve.

**Figure 9:**
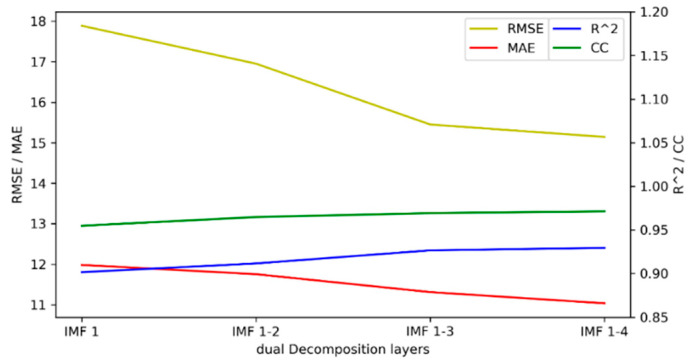
Performance metrics of the decomposition of different IMFs.

For the quantitative evaluation, the prediction performance is presented in Table 2. The performance metrics are also shown in Figure 9.

Figure 9 shows the change curves of RMSE, MAE, *R*^2^, and CC. As the components used for secondary decomposition increased, the RMSE and MAE gradually decreased, while *R*^2^ and CC gradually increased. The performance of the model achieved the optimal value when the IMF 1–4 components were subjected to dual decomposition. As can be observed from the results above, when the number of decomposition layers was four, the highest model accuracy was achieved. As the number of decomposition layers increased, the accuracy of the model gradually improved. When the number of decomposition layers was four, the best effect was achieved. Without decomposition, the RMSE was 18.2552, and the result of the four-layer decomposition was improved by 17.0461%. The MAE increased by 10.0290%, the *R*^2^ increased by 3.5655%, and the CC increased by 1.9523%.

#### 4.2.3. Comparison of Contrast Methods

In order to verify the proposed model, the RNN, LSTM, GRU, decomposition-ARIMA-GRU-GRU, and EMDCNN-GRU models were selected as the comparison models. The first three models represent classic machine learning and were constructed by three layers of simple RNN, LSTM, and GRU in Keras to simulate the input layer, hidden layer, and output layer, respectively. For the decomposition-ARIMA-GRU-GRU model, the period, trend, and residual components of the time series were generated by STL decomposition firstly. Then, the period and residual components were trained by the GRUs respectively, and the trend component was trained by the ARIMA model. Finally, all results were fused to form a prediction result. For the EMDCNN-GRU mode, the IMFs were generated by EMD decomposition, then all IMFs were divided into three groups by CNN. Each group was trained and predicted with the GRU, and the three prediction results were integrated.

Table 3 shows that the dual decomposition model obtained the smallest RMSE and MAE and the largest *R*^2^ and CC. Compared to RNN, LSTM, GRU, decomposition-ARIMA-GRU-GRU, and EMDCNN-GRU, our model achieved the best performance. The results show that the dual decomposition model can fully mine the data features and has a good predictive ability for nonstationary time series.

## 5. Discussion

In this study, a new prediction method for nonstationary time series data was proposed, combining dual VMD with a GRU network. On the basis of the first decomposition, dual decomposition is performed on subsequences with lower frequencies to fully reduce complexity and further improve the prediction accuracy. In this study, the criteria were designed to reasonably determine the decomposition layers and dual decomposition conditions in VMD.

Test 1 decomposed the original data with different layers. The optimal number of layers was consistent with the results obtained by the proposed criteria, which proves that the method is effective in determining the number of layers through information entropy. By decomposing the components obtained in Test 1, Test 2 quantitatively determined the components that needed to be further decomposed, which fully reduced the complexity and further improved the prediction accuracy based on the first decomposition, verifying the effectiveness of the method for determining the components of decomposition. Test 3 compared the RNN, LSTM, GRU, decomposition-ARIMA-GRU-GRU, and EMCDCNN-GRU models with our proposed method. Our dual decomposition model obtained the highest accuracy, which proves that dual decomposition is more effective.

The proposed model has outstanding prediction performance, but it takes a long time to build the model and takes up more computing resources, which we further investigate in the future. For data with different characteristics, we will try to combine multiple decomposition methods to make the method more effective. In addition, we will explore online learning methods to improve their applicability.

## 6. Conclusions

In this study, we investigated the issue of the prediction of nonlinear and nonstationary time series. A framework of deep prediction models with dual decomposition was proposed as the core method. In the proposed method, the quantitative criteria were explored to solve the feature extraction problem in VMD decomposition. The GRU deep model performed well in the prediction of subsequences. The experiments and results show that the first and second decomposition methods can reduce the difficulty of modeling and improve prediction performance. The criteria for the decomposition are effective and practical, which can help the automatic feature extraction of the time series. In the future, we will further investigate non-stationary time series forecasting [40,41,42,43,44], including optimization methods for decomposition models and multi-model fusion methods [45,46,47].

## Figures and Tables

**Table 1:** Prediction errors and times of different decomposition layers of data.

Layer	3	4	5	6	7
RMSE	34.4928	28.5985	22.2981	18.2552	18.8259
Time(s)	479.7493	573.4552	639.6171	734.5878	931.3032
**Layer**	**8**	**9**	**10**	**11**	**12**
RMSE	18.1537	18.0919	18.6195	18.5581	17.8504
Time(s)	1083.2551	1155.3032	1332.4787	1460.2881	1614.4549

**Table 2:** Prediction performance of the decomposition of different IMFs.

Different Decomposition Situations	RMSE	MAE	*R* ^2^	CC
First decomposition	18.2552	12.2644	0.8975	0.9527
IMF 1 to dual decomposition	17.8887	11.9822	0.9016	0.9548
IMF 1–2 to dual decomposition	16.9522	11.7535	0.9116	0.9648
IMF 1–3 to dual decomposition	15.4507	11.3111	0.9266	0.9693
IMF 1–4 to dual decomposition	15.1434	11.0344	0.9295	0.9713

**Table 3:** Comparison of methods.

Model	RMSE	MAE	*R* ^2^	CC
RNN	51.3712	36.2261	0.1884	0.4879
LSTM	52.9843	36.3533	0.1366	0.4512
GRU	49.9043	33.0322	0.2341	0.5175
Decomposition-ARIMA-GRU-GRU	49.6151	33.4335	0.2429	0.5136
EMDCNN-GRU	43.5485	33.7525	0.4167	0.6663
Dual Decomposition	15.1434	11.0344	0.9295	0.9713

## Data Availability

The data presented in this study are available upon request from the corresponding author.

## References

[1] Xu L., Li Q., Yu J., Wang L., Xie J., Shi S. (2020). Spatio-temporal predictions of SST time series in China’s offshore waters using a regional convolution long short-term memory (RC-LSTM) network. Int. J. Remote Sens..

[2] Liu L., Tianyao J., Mengshi L.I., Chen Z., Qinghua W. (2018). Short-term local prediction of wind speed and wind power based on singular spectrum analysis and locality-sensitive hashing. Mod. Power Syst..

[3] Hu H., Tang L., Zhang S., Wang H. (2018). Predicting the direction of stock markets using optimized neural networks with Google Trends. Neurocomputing.

[4] Bai Y., Wang X., Sun Q., Jin X., Wang X., Su T., Kong J. (2019). Spatio-Temporal Prediction for the Monitoring-Blind Area of Industrial Atmosphere Based on the Fusion Network. Int. J. Environ. Res. Public Health.

[5] Bai Y., Jin X., Wang X., Wang X., Xu J. (2020). Dynamic Correlation Analysis Method of Air Pollutants in Spatio-Temporal Analysis. Int. J. Environ. Res. Public Health.

[6] Yang Y., Bai Y., Wang X., Wang L., Jin X., Sun Q. (2020). Group decision-making support for sustainable governance of algal bloom in urban lakes. Sustainability.

[7] Rojas I., Valenzuela O., Rojas F., Guillén A., Herrera L.J., Pomares H., Marquez L., Pasadas M. (2008). Soft-computing techniques and arma model for time series prediction. Neurocomputing.

[8] Torres J.L., Garcia A., De Blas M., De Francisco A. (2005). Forecast of hourly average wind speed with ARMA models in Navarre (Spain). Sol. Energy.

[9] Tan Z., Zhang J., Wang J., Xu J. (2010). Day-ahead electricity price forecasting using wavelet transform combined with ARIMA and GARCH models. Appl. Energy.

[10] Bin L.I., Yi-Bin L.I. (2011). Chaotic time series prediction based on elm learning algorithm. J. Tianjin Univ..

[11] Wang F., Yu Y., Zhang Z., Li J., Zhen Z., Li K. (2018). Wavelet Decomposition and Convolutional LSTM Networks Based Improved Deep Learning Model for Solar Irradiance Forecasting. Appl. Sci..

[12] Zhang Y., Zhao Y., Kong C., Chen B. (2020). A new prediction method based on VMD-PRBF-ARMA-E model considering wind speed characteristic. Energy Convers. Manag..

[13] Xie T., Zhang G., Liu H., Liu F., Du P. (2018). A Hybrid Forecasting Method for Solar Output Power Based on Variational Mode Decomposition, Deep Belief Networks and Auto-Regressive Moving Average. Appl. Sci..

[14] Li G., Chang W., Yang H. (2020). Monthly Mean Meteorological Temperature Prediction Based on VMD-DSE and Volterra Adaptive Model. Adv. Meteorol..

[15] Cadzow J.A. (1983). ARMA Time Series Modeling: An Effective Method. IEEE Trans. Aerosp. Electron. Syst..

[16] Wang Y., Wang C., Shi C., Xiao B. (2018). Short-term cloud coverage prediction using the ARIMA time series model. Remote Sens. Lett..

[17] Garcia R.C., Contreras J., Akkeren M.V., Garcia J.B.C. (2005). A garch forecasting model to predict day-ahead electricity prices. IEEE Trans. Power Syst..

[18] Durbin J., Koopman S.J. (2012). Time Series Analysis by State Space Methods.

[19] Bai Y.-T., Wang X.-Y., Jin X.-B., Zhao Z.-Y., Zhang B.-H. (2020). A Neuron-Based Kalman Filter with Nonlinear Autoregressive Model. Sensors.

[20] Xue J., Zhou S.H., Liu Q., Liu X., Yin J. (2017). Financial time series prediction using 2,1rf-elm. Neurocomputing.

[21] Lin J., Cheng C., Chau K. (2006). Using support vector machines for long-term discharge prediction. Hydrol. Sci. J..

[22] Amjady N. (2006). Day-ahead price forecasting of electricity markets by a new fuzzy neural network. IEEE Trans. Power Syst..

[23] Yang C., Qiao J., Wang L., Zhu X. (2019). Dynamical regularized echo state network for time series prediction. Neural Comput. Appl..

[24] Che Z., Purushotham S., Cho K., Sontag D., Liu Y. (2017). Recurrent Neural Networks for Multivariate Time Series with Missing Values. Sci. Rep..

[25] Hochreiter S., Schmidhuber J. (1996). LSTM can Solve Hard Long Time Lag Problems. Adv. Neural Inf. Process. Syst..

[26] Fischer T., Krauss C. (2017). Deep learning with long short-term memory networks for financial market predictions. Eur. J. Oper. Res..

[27] Jin X., Yang N., Wang X., Bai Y., Su T., Kong J. (2020). Hybrid Deep Learning Predictor for Smart Agriculture Sensing Based on Empirical Mode Decomposition and Gated Recurrent Unit Group Model. Sensors.

[28] Ding M., Zhou H., Xie H., Wu M., Nakanishi Y., Yokoyama R. (2019). A gated recurrent unit neural networks based wind speed error correction model for short-term wind power forecasting. Neurocomputing.

[29] Cleveland R.B., Cleveland W.S., Mcrae J.E., Terpenning I. (1990). Stl: A seasonal-trend decomposition procedure based on loess. J. Off. Stat..

[30] Qin L., Li W., Li S. (2019). Effective passenger flow forecasting using STL and ESN based on two improvement strategies. Neurocomputing.

[31] Qiao W., Tian W., Tian Y., Yang Q., Wang Y., Zhang J. (2019). The Forecasting of PM2.5 Using a Hybrid Model Based on Wavelet Transform and an Improved Deep Learning Algorithm. IEEE Access.

[32] Gao X., Li X., Zhao B., Ji W., Jing X., He Y. (2019). Short-Term Electricity Load Forecasting Model Based on EMD-GRU with Feature Selection. Energies.

[33] Xu Y., Zhang J., Long Z., Chen Y. (2018). A Novel Dual-Scale Deep Belief Network Method for Daily Urban Water Demand Forecasting. Energies.

[34] Wang K., Niu D., Sun L., Zhen H., Xu X. (2019). Wind power short-term forecasting hybrid model based on ceemd-se method. Processes.

[35] Jin X., Yang N., Wang X., Bai Y., Su T., Kong J. (2019). Integrated Predictor Based on Decomposition Mechanism for PM2.5 Long-Term Prediction. Appl. Sci..

[36] Jin X.B., Yang N.X., Wang X.Y., Bai Y.T., Su T.L., Kong J.L. (2020). Deep hybrid model based on emd with classification by frequency characteristics for long-term air quality prediction. Mathematics.

[37] Niu W.-J., Feng Z.-K., Chen Y.-B., Zhang H.-R., Cheng C.-T. (2020). Annual Streamflow Time Series Prediction Using Extreme Learning Machine Based on Gravitational Search Algorithm and Variational Mode Decomposition. J. Hydrol. Eng..

[38] Yang Z., Ce L., Lian L. (2017). Electricity price forecasting by a hybrid model, combining wavelet transform, ARMA and kernel-based extreme learning machine methods. Appl. Energy.

[39] Zhang S., Lv J.N., Jiang Z., Zhang L. (2009). Study of the correlation coefficients in mathematical statistics. Math. Pract. Theory.

[40] Kong J., Wang H., Wang X., Jin X., Fang X., Lin S. (2021). Multi-stream Hybrid Architecture Based on Cross-level Fusion Strategy for Fine-grained Crop Species Recognition in Precision Agriculture. Comput. Electron. Agric..

[41] Kong J., Yang C., Wang J., Wang X., Zuo M., Jin X., Lin S. (2021). Deep-stacking network approach by multisource data mining for hazardous risk identification in IoT-based intelligent food management systems. Comput. Intell. Neurosci..

[42] Jin X.B., Zheng W.Z., Kong J.L., Wang X.Y., Bai Y.T., Su T.L., Lin S. (2021). Deep-learning Forecasting Method for Electric Power Load Via Attention-based encoder-decoder With Bayesian Optimization. Energies.

[43] Jin X.B., Zheng W.Z., Kong J.L., Wang X.Y., Zuo M., Zhang Q.C., Lin S. (2021). Deep-Learning Temporal Predictor via Bi-directional Self-attentive Encoder-decoder framework for IOT-based Environmental Sensing in Intelligent Greenhouse. Agriculture.

[44] Zheng Y.Y., Kong J.L., Jin X.B., Wang X.Y., Su T.L., Zuo M. (2019). Crop Deep: The Crop Vision Dataset for Deep-learning-based Classification and Detection in Precision Agriculture. Sensors.

[45] Jin X.B., Gong W.T., Kong J.L., Bai Y.T., Su T.L. (2022). PFVAE: A Planar Flow-Based Variational Auto-Encoder Prediction Model for Time Series Data. Mathematics.

[46] Jin X.B., Gong W.T., Kong J.L., Bai Y.T., Su T.L. (2022). A Variational Bayesian deep network with data self-screening layer for massive time-series data forecasting. Entropy.

[47] Jin X.B., Zhang J.S., Kong J.L., Su T.L., Bai Y.T. (2022). A Reversible Automatic Selection Normalization (RASN) Deep Network for Predicting in the Smart Agriculture System. Agronomy.

